# Supplementary material for: Differential development of antibiotic resistance and virulence between Acinetobacter species
Source: mSphere. 2024 Apr 5;9(5):e00109-24. doi: 10.1128/msphere.00109-24 (PMC11237425; doi:10.1128/msphere.00109-24)
Supplement: Supplemental Text and Figures — Texts S1 and S2 and Figures S1-S5. [file msphere.00109-24-s0001.docx]

**Supplementary Text and Figures**

**Supplementary Text** **S1**

Biofilm. Formation in static conditions and under laminar flow

Biofilm mass formed in static conditions was measured using a crystal violet biofilm assay as previously described in Ref. (1).

Biofilm formation under laminar flow was measured using the Bioflux system as previously described (2). After 48 hours, flow cells were pulsed at 5 dyne/cm^2^ for 5s to capture images of attached biofilm only. Experiments were performed with two technical and two biological replicates. Images were analysed using ImageJ (3), where the percentage of black pixels in the field of view was calculated as a proxy for biofilm growth. The median percentage coverage was then plotted on Graphpad Prism (4).

Antimicrobial susceptibility of established *A. baumannii* and *A. lwoffii* biofilms

The minimum biofilm eradication concentration of established static biofilms was determined using a peg lid model as previously described (5). Compounds tested included cefotaxime (Fisher #10084487), chlorhexidine (Sigma #C9394), ciprofloxacin (Fisher #13531640), meropenem (TCI Chemicals #M2279), oxacillin (Sigma, #O1002), tetracycline (Fisher #10460264), triclosan (Sigma, #72779) and rifampicin (Fisher #10533325).

**Supplementary Text S2**

Measurement of twitching motility

The twitching capacity of *A. baumannii* and *A. lwoffii* was determined as detailed previously (6). Sub-surface twitching halos were stained and measured using crystal violet.

Comparing the growth of *A. baumannii* and *A. lwoffii*

Growth in LB and male AB human serum (Merck, #H4522) was measured for 16 and 24 hours in a Fluostar Omega plate reader (BMG LabTech). The OD_600_ was measured at ten-minute intervals and a growth curve was plotted on Prism (v.9, Graphpad) (4). Mean generation time was computed using R package growthcurver (7) and comparative statistics performed in Prism. Human serum was used both fresh (NHS - normal human serum) and heat inactivated at 56°C for 1 hour (HIS - heat inactivated serum).

Survival in serum was also quantified. Briefly, cells were grown to mid-log phase and then diluted to 1x10^6^ CFU/mL. 10 μL of cells were added to 90 μL of either NHS or LB. Cells were incubated at 37°C with gentle rocking (20 rpm). 10 μL was removed at 0 minutes, 45 minutes, 1.5 hours, 3 hour and 24 hours and serially diluted to enumerate colony forming units (CFUs).

Strains were also grown in 5 mL synthetic wound fluid at 37°C with shaking for five hours and diluted to an OD of ~0.1. Wound fluid was created using a previously published method (8) in 24 well plates and the bacteria were statically incubated with humidity for 3 days at 37°C and visible growth was compared.

**Supplementary Fig. S1** A phylogenetic tree of MAFFT aligned *rep* genes from (9) and additional *A. lwoffii rep* genes from available complete genome sequences on NCBI (red). Arrows point to zoomed in areas of the tree to show additional *A. lwoffii rep* genes

**Supplementary Fig. S2** *A. lwoffii* forms less biofilm in static conditions than *A. baumannii,* but both species form similar levels of biofilm under laminar flow. a- total biofilm density grouped by species, b-Supp individual strain biofilm formation. Median values of three biological repeats plotted, along with interquartile range. Two-tailed Welch’s T-test proved significance when comparing the species, p <0.0001. c- percentage coverage of biofilm formed on a flow cell under laminar flow in the Bioflux model. Median coverage is plotted with interquartile range. d- number of biofilm associated genes found in whole genome sequences, whiskers show minimum and maximum values. Welch’s T test was used to compare the number of genes in either species after a random permutation test accounted for different sample sizes, p <0.0001. Pink - *A. baumannii*, yellow - *A. lwoffii*.

**Supplementary Fig. S3** Scanning electron micrographs of *A. baumannii* (AB) and *A. lwoffii* (AL) strains taken on an Apreo 2 (Thermo Fisher).

**Supplementary Fig S4** Growth curves of *A. baumannii* (pink)and *A. lwoffii* (yellow) in LB over twelve hours at 37°C. Optical density at 600_nm_ was measured every twenty minutes in a FLUOstar Omega Plate Reader.

**Supplementary Fig S5** The growth of *A. baumannii* and *A. lwoffii* in human serum

NHS = normal human serum, HIS = heat inactivated serum

**References:**

1. O’Toole GA, Kolter R. 1998. Initiation of biofilm formation in *Pseudomonas fluorescens* WCS365 proceeds via multiple, convergent signalling pathways: a genetic analysis. Molecular microbiology 28:449–461.

2. Holden ER, Yasir M, Turner AK, Charles IG, Webber MA. 2022. Comparison of the genetic basis of biofilm formation between *Salmonella Typhimurium* and *Escherichia coli*. Microbial Genomics 8:000885.

3. Rasband WS. ImageJ (1.52). National Institutes of Health, Bethesda, Maryland.

4. Miller JM. 2003. GraphPad PRISM (53).

5. Ravi NS, Aslam RF, Veeraraghavan B. 2019. A new method for determination of minimum biofilm eradication concentration for accurate antimicrobial therapy. *Acinetobacter baumannii*: Methods and Protocols 61–67.

6. Biswas I, Machen A, Mettlach J. 2019. In vitro motility assays for *Acinetobacter* species. *Acinetobacter baumannii*: Methods and Protocols 177–187.

7. Sprouffske K, Wagner A. 2016. Growthcurver: an R package for obtaining interpretable metrics from microbial growth curves. BMC bioinformatics 17:1–4.

8. Werthen M, Henriksson L, Jensen PØ, Sternberg C, Givskov M, Bjarnsholt T. 2010. An *in vitro* model of bacterial infections in wounds and other soft tissues. Apmis 118:156–164.

9. Lam MM, Koong J, Holt KE, Hall RM, Hamidian M. 2023. Detection and typing of plasmids in *Acinetobacter baumannii* using *rep* genes encoding replication initiation proteins. Microbiology Spectrum 11:e02478-22.
